# Emergency Medical Services and Primary Health Care linkage: a potential solution for effective emergency response in rural areas: the case of Ukraine

**DOI:** 10.3389/fpubh.2025.1691361

**Published:** 2025-10-02

**Authors:** Rafael Castro-Delgado, Manisha Panta Bhandari, Radha Subedi Acharya

**Affiliations:** ^1^Faculty of Medicine and Health Sciences, University of Oviedo, Oviedo, Spain; ^2^Health Service of the Principality of Asturias (SAMU-Asturias), Oviedo, Spain; ^3^Health Research Institute of the Principality of Asturias (Research Group on Prehospital Care and Disasters, GIAPREDE), Oviedo, Spain

**Keywords:** Emergency Medical Services, Primary Health Care (PHC), emergency response, health management, health policy

## Abstract

Ukraine’s emergency medical system faces significant challenges, particularly in rural and conflict-affected areas, where gaps in coordination, infrastructure, and access to care persist. Despite investments and reforms, the fragmentation between Emergency Medical Services (EMS) and Primary Health Care (PHC) has limited the effectiveness of emergency response, especially in rural areas. This viewpoint proposes the integration of EMS and PHC as a strategic solution to enhance response capacity, reduce delays, and improve health outcomes in rural populations. Drawing on the successful model from the Asturias region in Spain, an example of PHC network for WHO and where PHC is embedded in emergency response pathways, the article outlines a feasible roadmap for Ukraine that may be followed by other countries to improve emergency response in rural areas. Key elements include the establishment of joint protocols, integrated communication systems, standardized referral pathways, and targeted personnel training. Following European Union regulations, the adoption of a single emergency number (112) is also discussed as a critical step toward improved interagency coordination. By aligning PHC infrastructure with EMS operations, Ukraine can strengthen its emergency care system and ensure equitable access to timely medical interventions across its territory.

## Highlights

- Emergency care in rural and remote areas is a challenge for health policy makers
- Emergency Medical Services (EMS) has traditionally been developed separately from Primary Health
- Care network
- EMS and PHC linkage in remote areas may be an efficient solution to manage emergencies
- Asturias model in Spain may be replicable model to follow
- Ukrania is used as a theoretical example to implement this model

## Introduction

In Ukraine, Emergency Medical Services (EMS) are a fundamental part of the healthcare system, led by the Cabinet of Ministers and Ministry of Health and coordinated by the Centres for Emergency Medical Care and Disaster Medicine (CDM).

The system operates nationwide through a comprehensive network of emergency call centres and sub centres, accessible through emergency numbers 103 and 112 (1). The country has faced significant challenges, particularly due to the war with Russia in 2014 and 2022 which has severely impacted the implementation of EMS legislation and created critical service gaps (2, 3), particularly in rural and conflict-affected areas (4). In 2024, Ukraine’s health system operated under extraordinary pressure due to the ongoing war. The resilience of health workers, institutions, and communities ensured access to care despite the war’s impact. Emergency Medical Services (EMS) and Emergency Medical Teams (EMTs) played an essential role in improving Ukraine’s emergency system by enhancing pre-hospital care, hospital readiness, mass casualty response, and national coordination (5). However, research emphasize that rural areas of Ukraine, continue to face challenges such as inadequate healthcare infrastructure, a shortage of medical personnel, and difficulties in accessing emergency care, despite reforms and investments. These issues contribute to higher rates of illness and death in rural populations, particularly during crises like war (6).

## Emergency response system and challenges

Emergency response systems in Ukraine frequently face challenges in coordination (7). Despite the presence of disaster medicine units, civil defence teams, and emergency services, effective collaboration during crises is often lacking (8). Dispatch services are overwhelmed, interagency protocols are unclear, and many ambulances have been damaged or destroyed (9), creating shortages in cross-country ambulances and response capabilities in conflict-affected and remote areas. As a result, there may be coverage gaps, delayed responses, and compromised patient care (10). Many hospitals lack dedicated emergency departments and standardized triage systems and have secondary referrals arranged in an *ad hoc* manner (10). Additionally, as the study conducted by WHO notes, Ukraine is facing severe staff shortages, insufficient medical equipment, and limited financial resources (10).

This demonstrates the critical need for systematic improvements to Ukraine’s emergency response coordination and infrastructure especially in the rural areas, as mandated by the Law of Verkhovna Rada of Ukraine No. 7117, On Improvement of Accessibility and Quality of Health Care Services in Rural Areas in Ukraine. Research evidence supports the benefits of integrated care models. Integrated care models, particularly for patients with acute medical emergencies, have been found to provide potential benefits in reduced mortality, readmission, adverse events, length of stay, and quality of life (11).

## Current PHC system challenges in Ukraine

Ukraine has a network of Primary Health Care (PHC) centres, but these centres are underutilized, and this reality cannot be overlooked. This is particularly evident in the rural areas, where most physicians and health care providers are not willing to work there. Rural healthcare posts across Ukraine have no access to water supply, sanitation services, and the majority of rural outpatient clinics have no sanitary units at all (12). Additionally, Mobile Health Units (MHUs) are poorly integrated into the primary healthcare framework, which lacks essential health services for Ukrainians.

The importance of utilizing existing PHC infrastructure cannot be overstated, particularly given Ukraine’s legislative recognition (Law no. 7117), which directly supports the argument for EMS-PHC integration in emergency response by addressing the foundational infrastructure and staffing issues that make such integration both necessary and feasible. Recent evidence shows that strong primary care is associated with better population health, lower rates of unnecessary hospitalizations and relatively lower socioeconomic inequality (13).

Ukraine has continued to strengthen its Primary Health Care system recently, despite wartime challenges. With World Health Organization (WHO) assistance, the country has established new healthcare centres in eastern regions and deployed mobile medical teams that reached over 24,000 people in isolated areas. Financial reforms have directed more resources to frontline and rural facilities, ensuring continued care for displaced persons and vulnerable communities (14). Enhancement efforts, for instance, are infrastructure improvements across hundreds of health facilities, distribution of medical equipment, and training programmes for healthcare professionals. Ukraine has also successfully integrated specialized services such as mental health support and rehabilitation into primary care. PHC services are providing care for personnel with noncommunicable diseases (NCDs) as well as addressing urgent humanitarian needs.

However, instead of strengthening the Primary Health Care centre (PHC system) itself, in Ukraine’s Primary Health Care (PHC services) are provided through Outreach Health Units (OHUs) and EMTs, which reach communities with limited or no access to care, providing life-saving interventions, trauma stabilization, and PHC services. OHUs, which receive essential equipment and medications from the WHO, provide medical care in remote locations. The Units deliver PHC services in coordination with the National Health Service of Ukraine (NHSU) and the Programme of Medical Guarantees (PMG). Efforts have also been made to develop a plan for PHC infrastructure development and restoration through a comprehensive needs assessments conducted in 41 PHC centres in Kharkiv oblast and a similar needs assessment is continuing (14).

Therefore, prioritizing reforms and interorganizational integration is a practical necessity. While this transformation is ongoing, this report makes a strong recommendation to improve EMS-PHC collaboration in emergency response, which will enable Ukraine to solve various critical problems related to emergency service delivery. Various successful examples exist of seamless coordination along the patient care pathway, bridging EMS with broader health and social systems (15).

## Asturian model of EMS – a potential solution

**Figure 1:**
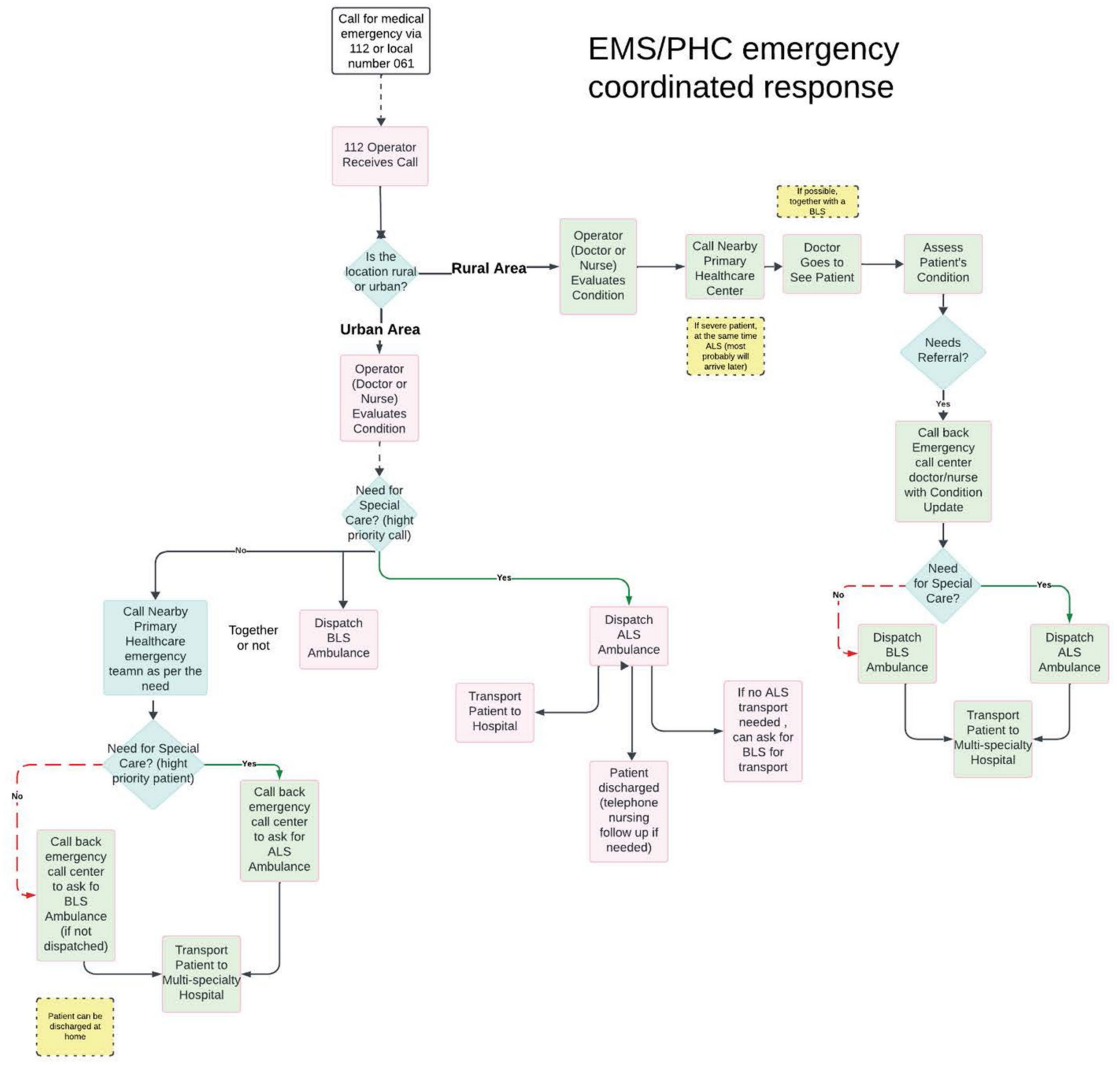
Asturian model of system integration, which explains how EMS and PHC centres are interrelated in providing care, especially in rural areas.

Primary Health Care (PHC) in Spain has, as many other countries, challenges to be faced (16). Even though, Asturias Health System in Spain has been recognized by the WHO Primary Health Care Demonstration Platform (17). In Asturias PHC network in integrated in emergency response in rural areas, but also in urban areas for low priority emergencies, which can serve as an excellent blueprint (18). This model integrates primary healthcare (PHC) centres into the EMS response network and establishes standardized care protocols across all levels (Figure 1).

## Urban area response system

The process follows a direct, streamlined path when a 112 doctor or nurse receives an emergency call from an urban area. An operator immediately evaluates the patient’s condition by means of a telephone call. Based on this evaluation, they assess whether there is a need for special care. If yes, they directly dispatch an ALS ambulance. If no, they have two simultaneous options: they can either call the nearby Primary Health Care emergency team depending on the need AND/OR dispatch a BLS ambulance together with a team or not. If special care is needed after this, an Advanced Life Support (ALS) ambulance is dispatched to work in coordination with the PHC team. Meanwhile, for less severe cases, a Basic Life Support (BLS) ambulance is sent to transport the patient to hospital. If no ALS transport is available, they can request BLS transport. The patient can also be discharged to home with telephone EMS nursing follow-up if needed.

## Rural area response system

The rural response system follows a more comprehensive pathway involving multiple healthcare providers. When a call is received from a rural area, the doctor or nurse first evaluates their condition, in a similar way as the urban process. However, they contact the nearby Primary Health Care (PHC) centre instead of dispatching an ambulance immediately. A doctor from this centre, with BLS or not, then physically attends the patient and conducts an in-person assessment. After evaluation, if the patient requires a referral, the operator is called again with an update on the patient’s condition. Based on this updated assessment, the EMS doctor at the emergency call centre, in agreement with the PHC doctor, determines whether special care is required. An ALS ambulance is dispatched for cases requiring advanced medical support; otherwise, a BLS ambulance is sent. The final step involves transporting the patient to a multi-speciality hospital, ensuring access to comprehensive medical care despite the rural location. For some cases, the patient can be discharged to home directly after assessment.

Each PHC centre provides regular appointments, consultations, care, and home visits. In emergencies, the network of emergency centres, which operates through the 112 number system. This model employs various ambulances, from basic transport ambulances (A1) to fully equipped mobile intensive care units (Class C). This integrated system exemplifies a commitment to accessibility and coordination, ensuring that individuals receive the appropriate care in emergencies (19).

**Figure 2:**
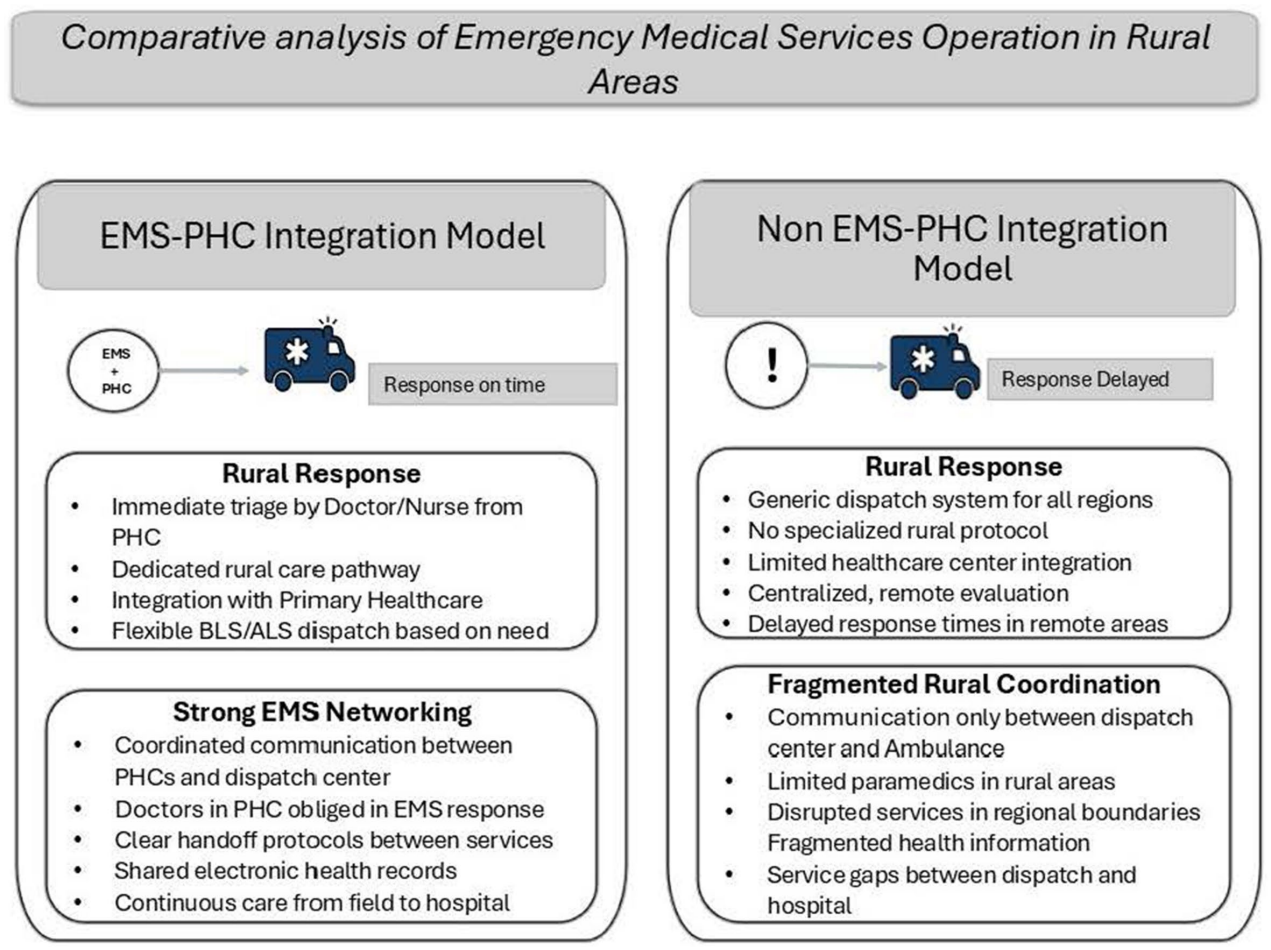
Key distinction between the integrated and non-integrated system.

The key distinction lies in how PHC centres are integrated (Figure 2). In Urban areas specifically, involvement of PHC in each emergency case is based on need as determined by the initial assessment and advanced resources availability, whereas in rural areas, any response to emergency calls initially involves PHC. This is to ensure timely response to any medical emergencies.

## Benefit of including the Asturian (EMS-PHC integration) model

Implementing this model enables Ukraine to improve its response times to rural populations without the need to develop more EMS stations. Moreover, establishing clear procedures for secondary referrals is vital for ensuring that patients receive appropriate follow-up care. To successfully implement this integration, a clear, organized strategy needs to be developed. This specific first step is to establish protocols with well-defined roles, responsibilities, and communication channels for interaction between PHC and EMS. Secondly, establishment of an integrated communication mechanism between the PHC personnel and EMS and personnel training is necessary for effective integration. Lastly, there is the need to establish criteria for referrals, standard methods for communication and to implement feedback mechanisms for continuous quality improvement.

In addition to this, adopting a single emergency number, “112” by Ukraine represents another significant step toward system integration (20). This change aligns with European standards and can simplify the process for citizens seeking emergency assistance by facilitating better coordination among different emergency services, including police, fire, and medical responders (21, 22).

## Conclusion

The Emergency Medical Services (EMS) in Ukraine certainly struggle with urgent issues related to coordination, standardization, and resource allocation. This can be best achieved through integration of PHC centres with EMS, providing a standard protocol. There are already various efforts that have been made to strengthen primary care services through introducing and mobilizing various services. Instead, integrating PHC itself in the EMS system will solve many existing problems Ukraine is facing in service delivery.

This integration strategy is supported by extensive evidence from numerous studies that have demonstrated improvements in cost-effectiveness, system efficiency, and patient outcomes. Adopting this evidence-based strategy represents a step toward creating a more robust and efficient emergency healthcare system for Ukraine.

These strategies are not simply recommendations, they are necessary actions that, along with comprehensive staff training and clear referral pathways, will significantly improve Ukraine’s EMS, particularly in rural areas.

## Data Availability

The data analyzed in this study is subject to the following licenses/restrictions: not available. Requests to access these datasets should be directed to castrorafael@uniovi.es.
